# Chromosomal confirmation of the separate species status of *Melanotus villosus* (Geoffroy in Fourcroy, 1785) and *M. castanipes* (Paykull, 1800) (Coleoptera, Elateridae)

**DOI:** 10.3897/compcytogen.20.215012

**Published:** 2026-11-06

**Authors:** Robert B. Angus, Adeline Maury

**Affiliations:** 1 Department of Life Sciences (Insects), The Natural History Museum, London SW7 5BD, UK Department of Life Sciences (Insects), The Natural History Museum London United Kingdom; 2 Department of Biological Sciences, Royal Holloway University of London, Egham, Surrey TW20 0EX, UK Department of Biological Sciences, Royal Holloway University of London Egham United Kingdom

**Keywords:** Chromosomes, Elateridae, karyotypes, *Melanotus castanipes*, *Melanotus villosus*, species confirmation

## Abstract

Karyotypes, with C-banding, are given for *Melanotus villosus* (Geoffroy in Fourcroy) and *M. castanipes* (Paykull). The preparations are from mid-gut and gonads of adults, and mid-gut and testis of larvae. *M. villosus* has 2n = 18 + X (♂), 18 + XX (♀), while *M. castanipes* has 2n = 20 + X (♂), 20 + XX (♀). The X chromosome of *M. villosus* is the smallest in the nucleus, while in *M. castanipes* it is among the largest. Heterochromatin is confined to small centromeric C-bands in both species. The differences between the karyotypes of the two species in both total number chromosomes present and in particular the size of their X chromosomes are sufficient to confirm that two distinct species are involved.

## Introduction

The identities and number of *Melanotus* Eschscholtz, 1829 species associated with rotting trees have been a subject of confusion and dispute, not least among British Coleopterists, for many years. An outline of this is given by Howard Mendel (2024: 320–321). In the early 2000s, while a curator in the Coleoptera section of the Natural History Museum, he suggested to R.B.A. that a chromosomal investigation of these species could provide useful information, especially in the light of chromosomal differences between *Melanotus* species noted by Smith (1953) and Smith and Virkki (1978). As a result of this R.B.A. offered the topic to Adeline Maury for her third-year undergraduate research project, and the following account is based on her work for this project.

The objective of this study is to establish the status of the two British *Melanotus* species, *M. villosus* (Geoffroy in Fourcroy, 1785) and *M. castanipes* (Paykull, 1800). Morphological characters useful for distinguishing the two species are given by Mendel (2024) and Whitehead (2023).

## Material and methods

### M. castanipes

1 adult + 4 larvae, Suffolk, Butley, leg. H. Mendel.

6 larvae + 2 adults, Surrey, Runnymede, Cooper’s Hill, leg. R.B.A.

2 larvae, Hampshire, New Forest, Denny Wood, leg. R.B.A.

### M. villosus

10 larvae + 1 adult, Surrey, Richmond Park, leg. R.B.A.

1 larva, Berkshire, Windsor Deer Park, leg. R.B.A.

Methods for chromosome preparation and C-banding were as described by Angus (2006). Preparations were photographed under oil immersion, using a precision interference filter to give a monochromatic green light (for maximum contrast with the purple Giemsa-stained chromosomes) on to Agfa Copex Pan microfilm. Prints were made at a magnification of 3000X. Chromosomes were cut out and arranged as karyotypes, then scanned into a computer using using Adobe Photoshop.

## Results

Very early in the work it was clear that two distinct karyotypes were present. The karyotype obtained from Howard Mendel’s Suffolk material was taken as *H. castanipes*, and the other as *M. villosus*.

**Figure 1.**
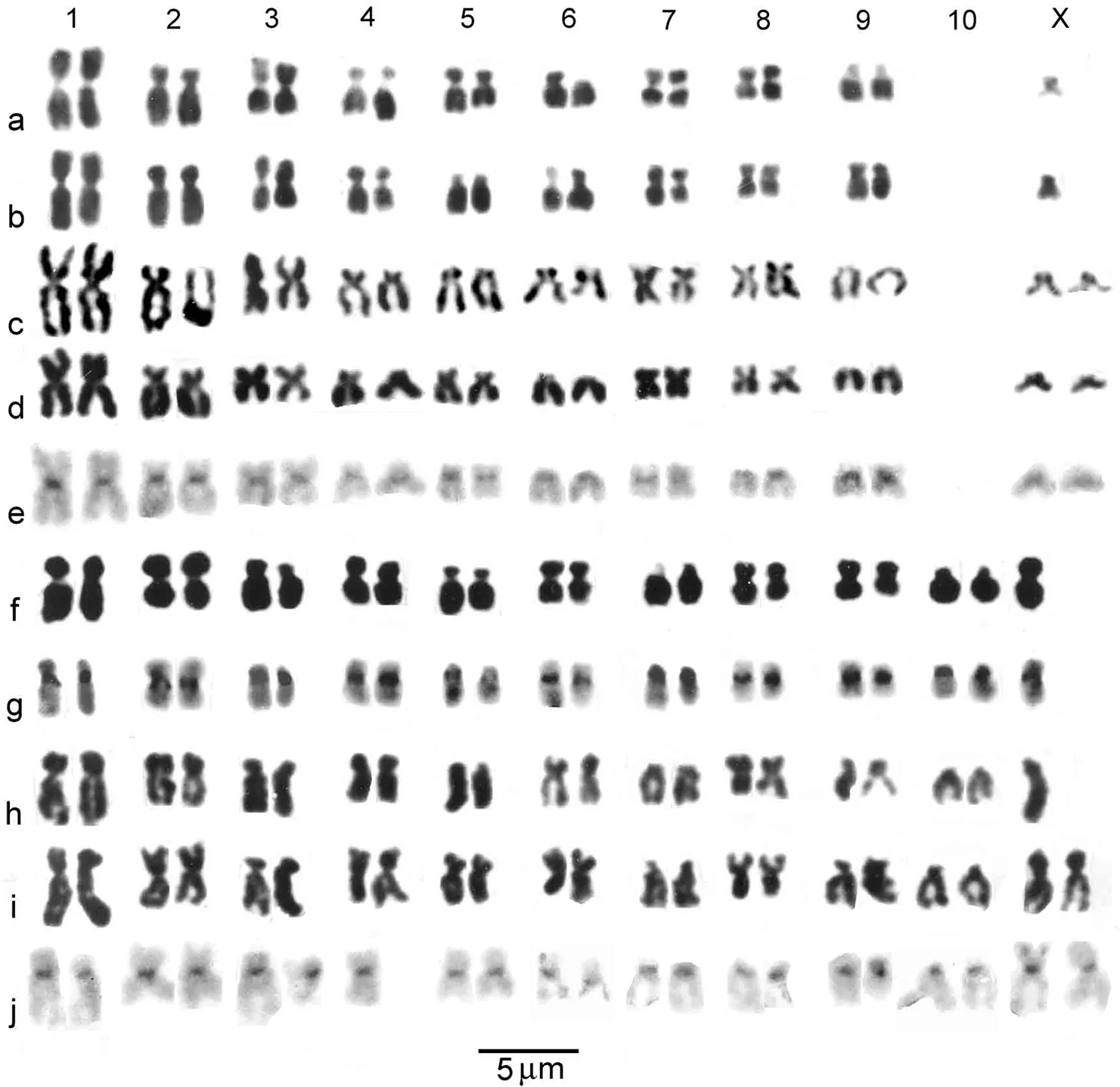
*Melanotus villosus* and *M. castanipes*. Karyotypes from mitotic chromosomes from larvae. **a–e**. *M. villosus*. **a, b**. ♂♂, Richmond Park, testis; **c–e**. ♀♀, Richmond Park, mid-gut; **d, e**. The same nucleus; **d**. Plain; **e**. C-banded. **f–j**. *M. castanipes*. **f, g**. ♂, Denny Wood, testis, the same nucleus; **f**. Plain; **g**. C-banded; **h**. Butley, ♂, mid-gut; **i**. ♀, Butley, mid-gut; **j**. ♀, Butley, mid-gut, C-banded, 1 replicate of autosome 4 missing. Scale bar: 5 μm.

The karyotypes are shown in Fig. 1.

Relative Chromosome Lengths (RCL, the length of each chromosome as a percentage of the total haploid chromosome length in the nucleus) and Centromere Indices (CI, the length of the short arm as a percentage of the total length of the chromosome) are given in Table 1. These terms were introduced by Levan et al. (1964) and Sumner (2003) gives a succinct and clear account of CIs which are classified as metacentric (m, CI 46–50), submetacentric (sm, 26–45) and subacrocentric (sa, 16–25). The 95% confidence limits of both the RCLs and the CIs were obtained from one-sample t-tests using the IBM SPSS.

**Table 1.** Relative Chromosome Lengths and Centromere Indices of the chromosomes.

***M. castanipes***											
**chromosome**	**1**	**2**	**3**	**4**	**5**	**6**	**7**	**8**	**9**	**10**	**X**
Mean RCL	14.63	11.40	11.04	10.71	9.87	9.61	8.87	8.51	8.21	7.33	11.81
95% confidence intervals	14.03–15.22	10.93–11.87	10.63–11.44	10.20–11.21	9.28–10.45	9.20–10.21	8.46–9.27	8.07–8.95	7.70–8.71	6.95–7.71	9.92–13.69
N	20	20	20	20	20	20	20	20	20	18	11
Mean CI	26.2	35.95	18.14	29.89	29.02	23.46	14.49	29.04	30.33	12.60	30.33
95% confidence intervals	24.29–28.10	33.72–38.14	16.07–20.22	28.0–31.78	26.57–31.47	20.93–26.00	12.99–15.99	25.29–32.79	27.73–32.93	10.38–14.82	26.29–35.37
N	20	20	20	20	20	20	20	20	20	18	11
***M. villosus***											
Mean RCL	19.79	15.2	12.04	10.68	9.67	8.58	8.44	7.84	7.21		5.09
95% confidence intervals	18.87–20.71	14.55–15.84	11.44–12.64	10.14–11.22	9.18–10.15	8.11–9.04	7.97–8.90	7.47–8.20	6.78–7.64		4.61–5.56
N	20	19	20	20	20	20	20	19	20		12
Mean CI	38.5	25	39	26.4	27.6	32.2	23.3	18.9	27.8		27.4
95% confidence intervals	36.2–40.8	25–27.1	39–41.7	24.3–28.5	24.9–30.3	28.5–36	18.9–27.7	15.7–22.1	25.1–30.5		25.6–29.2
N	20	19	20	20	20	20	20	19	20		12

It can be seen that there is very little agreement between the RCLs of *M. villosus* and *M. castanipes*. Only in the case of chromosomes 7, 8 and 9 does the result appear to be similar (95% confidence limits overlapping). It can also be seen that there is no agreement between the CIs of the two species, although both species have most of their chromosomes submetacentric. The long, almost metacentric, autosome 1 of *M. villosus* is distinct. The C-bands of both species are small and localised, so there is no extensive development of constitutive heterochromatin, which might account for size differences. Therefore the differences in relative lengths between some of the chromosomes of the two species would have to be the result of translocations of material between chromosomes. The X-chromosome is very different between both species, which would prevent normal pairing at meiosis in any hybrids, resulting in their infertility.

## Discussion

The genomes of the two *Melanotus* species both have an X0 sex determining system, that is, the males have a single X chromosome but no Y chromosome, while the females have two X chromosomes. This arrangement is fairly unusual in the suborder Polyphaga (to which the Elateridae belong), though it is the norm in the Adephaga.

Sivell et al. (2024), as part of the Darwin Tree of Life project, give the genome of *M. villosus* with 10 chromosomes including the X which is the smallest in the nucleus.

Undergraduate research projects are inevitably carried out during the winter (Autumn and Spring terms), and, although adults can be collected in the winter, we found little chromosomal activity in their guts and gonads, and none at all in material collected after early November. Fortunately the guts of the larvae showed active cell division, and we were able to use the karyograms to identify the species.

Smith and Virkki (1978) give chromosomal data on 10 species of *Melanotus*. For eight of these they give the male diploid number as 19, comprising nine pairs of autosomes and one X chromosome. However, for *M. castanipes* and one unidentified Canadian species they give the male diploid number as 18, with no further information. The data on these two species are from early work by Smith (1953), where they are listed as from Smith’s unpublished data. One way to account for this reduction to an even chromosome number in males is to suggest fusion of the X chromosome with an autosome, giving a neo-XY sex determining mechanism. However, *M. castanipes* has a male diploid number of 21 so whatever the explanation of Smith’s data, they do not refer to *M. castanipes*.

The main conclusion from this work is that *M. castanipes* and villosus are definitely different species.

## Additional information

### Artificial Intelligence (AI) use

The authors accept full responsibility for the content of the manuscript, including the disclosure of any use of AI.

No AI tools were used in the preparation of this manuscript.

### Author ORCIDs

R.B. Angus https://orcid.org/0000-0002-3860-5617

### Data availability

All of the data that support the findings of this study are available in the main text.
